# Genetic characterization and population structure of Indian rice cultivars and wild genotypes using core set markers

**DOI:** 10.1007/s13205-016-0409-7

**Published:** 2016-03-26

**Authors:** Malathi Surapaneni, Divya Balakrishnan, Sukumar Mesapogu, Addanki Krishnam Raju, Yadavalli Venkateswara Rao, Sarla Neelamraju

**Affiliations:** ICAR-National Professor Project, Indian Institute of Rice Research, Hyderabad, India

**Keywords:** Diversity, PCA, Population structure, SSRs, Wild rice

## Abstract

Genetic diversity among 23 rice genotypes including wild species and cultivars of *indica*, *japonica*, *aus* and aromatic type was investigated using 165 genomewide core set microsatellite (SSR) markers. This genotypic characterization was undertaken to know the genetic similarity among the parental lines to be used in developing a set of chromosome segment substitution lines. In all, 253 alleles were identified using 77 polymorphic SSRs, and polymorphism information content ranged from 0.31 to 0.97 with a mean of 0.79. Cluster analysis grouped the genotypes into three clusters at a genetic similarity of 0.26–0.75. Wild accessions grouped together in cluster-I, *indica* cultivars formed cluster-II, and aromatic, *japonica* and *aus* types came under cluster-III. Principal component analysis also showed similar results. The genotypic data was analyzed using STRUCTURE, and genotypes were grouped into four populations. RM1018 on chromosome 4, RM8009 on chromosome 7, and RM273 on chromosome 12 amplified alleles specific to wild accessions. The information obtained from core set markers would help in selecting diverse parents including wild accessions and for tracking alleles in mapping or breeding populations.

**Electronic supplementary material:**

The online version of this article (doi:10.1007/s13205-016-0409-7) contains supplementary material, which is available to authorized users.

## Introduction

Rice (*Oryza sativa* L.) is one of the most extensively cultivated cereal crops in the world spreading across a wide range of geographical, ecological and climatic regions. High genotypic and phenotypic diversity exists, and about 1,20,000 different accessions are reported in rice as a consequence of varied adaptations (Das et al. 2013). Large collections of rice germplasm consisting of cultivars of *indica*, *japonica* and *javanica* sub-species along with landraces are conserved in global gene banks (Khush 1997; Tanksley and McCouch 1997; Lu et al. 2005; Garris et al. 2005). Wild accessions of rice are potential reservoir of valuable genes that could be exploited for crop improvement programs (Brar and Khush 1997). There was a significant reduction in genetic diversity during the course of domestication from wild relatives to cultivated rice (Brar and Khush 2003; Sun et al. 2001). In spite of the richness of genetic resources, only a small proportion has been utilized in breeding programs, resulting in high genetic similarity in commercial rice cultivars (Das et al. 2013). To meet the future requirements of food, the yield potential of the varieties has to be improved, but it is difficult due to narrow genetic base of the popular varieties. To break the yield plateau, it is essential to broaden the genetic base of the varieties by introgressions from distant or newer gene pools. Assessment and introgression of the available genetic diversity in germplasm resources is required for detection of novel genes or QTLs for agronomically important traits.

Knowledge of the genetic diversity and genetic relationships between germplasm accessions is the basic foundation for crop improvement programs (Thomson et al. 2008). Domestication and artificial selection pressure changed the genome compositions and population structure of the available germplasm resources (Huang et al. 2012; Yadong et al. 2012). In depth analysis of population structure is required before initiating any breeding program to get targeted improvement in the traits. There are several ways for estimation of diversity in germplasm viz, evaluation of phenotypic variation, biochemical and DNA polymorphisms. However, both phenotypic and biochemical characterizations are not much reliable because they are environmentally influenced, labor demanding, numerically and phenologically limited, but DNA-based molecular markers are ubiquitous, repeatable, stable and highly reliable (Virk et al. 2000; Song et al. 2003). Among the several classes of available DNA markers, microsatellite or simple sequence repeat (SSR) markers are considered the most suitable due to their multi-allelic nature, high reproducibility, codominant inheritance, abundance and extensive genome coverage (Orjuela et al. 2010). In rice, high-density SSR map with genome coverage of approximately 2 SSRs per centimorgan (cM) (McCouch et al. 2002) and a universal core genetic map (UCGM) (Orjuela et al. 2010) were reported. These maps can help in accurately assessing genomewide diversity. Many researchers evaluated genetic diversity of Indian rice germplasm using other SSRs, but SSRs from UCGM have not been used for studying diversity previously (Saini et al. 2004; Jain et al. 2004; Ram et al. 2007; Sundaram et al. 2008; Kumar et al. 2010; Sivaranjani et al. 2010; Vanniarajan et al. 2012; Yadav et al. 2013). Studies were also conducted globally on classifying rice genotypes based on their genetic diversity and population structure using molecular markers (Garris et al. 2005; Zhao et al. 2010; Jin et al. 2010; Li et al. 2010; Zhang et al. 2011; Wang et al. 2014; Raimondi et al. 2014; Shinada et al. 2014). Most of the diversity studies in rice mainly focused on clustering within cultivated rice group and only few studies were reported using accessions from wild rice species in diversity analysis (Ni et al. 2002; Ravi et al. 2003; Caicedo et al. 2007; Ram et al. 2007). The present study was aimed at investigating the diversity and relationship among selected wild rice accessions and cultivars using SSR markers for the identification of diverse genotypes and polymorphic markers for further utilization in inter-specific breeding programs, and for the development CSSL population from elite/wild crosses.

## Materials and methods

### Plant material and DNA isolation

In this study, 23 rice genotypes were used including 4 wild accessions, 14 high yielding *indica* popular rice varieties along with two basmati varieties, 1 tropical *japonica*, 1 temperate *japonica* and 1 *aus* type from *indica* subspecies (Table 1). The materials were obtained from germplasm collections from the crop improvement section, Indian Institute of Rice Research, Rajendra Nagar, Hyderabad. The four wild accessions were selected based on their high photosynthesis rate (Kiran et al. 2013), and obtained from Physiology Department, IIRR. Thirty seeds per genotype were germinated in petri dishes and seedlings were transplanted in individual pots in green house. Young leaves (20 days after transplantation) were collected and genomic DNA was isolated using CTAB method (Doyle and Doyle 1987). Purity and concentration of DNA was monitored using Nano Drop ND-1000 Spectrophotometer (Wilmington, USA).

**Table 1:** List of genotypes used in the study

S. no	Genotype		Pedigree	Year of release
1	*O. rufipogon*-*A*	wild	(Acc No: CR100267)	–
2	*O. rufipogon*-*B*	wild	(Acc No: CR100309)	–
3	*O. nivara*-*C*	wild	(Acc No: CR100008)	–
4	*O. nivara*-*D*	wild	(Acc No: CR100097)	–
5	Swarna	*indica*	Vasistha/Mahsuri	1982
6	MTU1010	*indica*	Krishnaveni/IR64	2000
7	MTU1081	*indica*	Ajaya/BPT5204	–
8	Rasi	*indica*	TN-1/T-141	1977
9	Dhanrasi	*indica*	B32 Sel.4/*O. rufipogon*	2002
10	Krishnahamsa	*indica*	Rasi/Finegora	1997
11	Varadhan	*indica*	Swarna/IET9314//BR327-36	2008
12	Vandhana	*indica*	C-22/Kalakeri	2002
13	Tulasi	*indica*	Rasi/Finegora	1988
14	Tellahamsa	*indica*	HR12/TN1	1971
15	Sahbhagi Dhan	*indica*	IR55419-04*2/WayRarem	2009
16	IR64	*indica*	IR5657-33-2-1/IR2061-465-1-5-5	1991
17	Jaya	*indica*	TN-1/T-141	1968
18	BPT5204	*indica*	TN-1/F1(Mahsuri/GEB24)	1986
19	PusaBasmati-1	*indica,* aromatic	Pusa-150/Kernal Local	1989
20	Basmati 370	*indica*, aromatic	Pure line selection Dehraduni Basmati landrace	1973
21	Moroborekan	*tropical japonica*	NA	NA
22	Nipponbare	*temperate japonica*	NA	NA
23	N22	*indica*, *aus*	Selection from Rajbhog	1978

### Genotyping

165 SSR core set primers (Orjuela et al. 2010), which are evenly distributed across 12 chromosomes with a distance ranging between 2 and 3.5 Mbp were used to estimate the genetic relatedness among 23 genotypes. PCR reactions were carried out in Thermal cycler (Veriti PCR, Applied Biosystems, USA) with the total reaction volume of 10 μl containing 15 ng of genomic DNA, 1X assay buffer, 200 μM of dNTPs, 1.5 mM MgCl_2_, 10 pmol of forward and reverse primer and 1 unit of *Taq DNA polymerase* (Thermo Scientific). PCR cycles were programmed as follows: initial denaturation at 94 °C for 5 min followed by 35 cycles of 94 °C for 45 s, 55 °C for 30 s, 72 °C for 45 s, and a final extension of 10 min at 72 °C. Amplified products were resolved on 4 % metaphor agarose gels prepared in 0.5 X TAE buffer and electrophoresis was conducted at 120 V for 2 h. Gels were stained with ethidium bromide and documented using gel documentation system (Alpha imager, USA). Only clear and unambiguous bands of SSR markers were scored. Amplified fragments were scored for the presence (1) or absence (0) of the corresponding band among the genotypes, and formed a data matrix comprising ‘1’ and ‘0’.

### Data analysis

Polymorphism information content (PIC) of each SSR marker was computed according to the formula: PIC = 1 − ΣPi^2^ − ΣΣPi^2^ Pj^2^, where ‘i’ is the total number of alleles detected for SSR marker and ‘Pi’ is the frequency of the *i*th allele in the set of genotypes investigated and *j* = *i* + 1 (Botstein et al. 1980). Genetic relatedness among the genotypes was calculated using unweighted pair group method with arithmetic averages algorithm (UPGMA) by the program NTSYSpc 2.02i (Rohlf 1989). The unweighted neighbour joining (UNJ) cluster analysis followed by bootstrap analysis with 100 permutations was carried out using DARwin ver. 5.0.145 software (Perrier et al. 2003; Perrier and Jacquemoud-Collet 2006). Principal component analysis (PCA) was also done using the subroutine EIGEN using NTSYSpc 2.02i (Rohlf 1989). The genotypic data was analyzed using the graphical genotypes software (GGT 2.0) (Van Berloo 2008). The population structure was inferred through the model-based program, STRUCTURE 2.3.3 and was implemented with a burn in period of 5000 and run length of 50,000 markov chain monte carlo number (MCMC) replications for a population range from *K* = 2 to  10 (Pritchard et al. 2000). Final *K* value was determined using the Evannos Δ*K* method and Ln probability data was used to detect presence of genetically distinct populations using graphical approach (Evanno et al. 2005).

## Results and discussion

Assessment of genetic variation in germplasm collections is essential for the efficient conservation, characterization and utilization of biodiversity. A total of 165 core set SSRs from UCGM (Orjuela et al. 2010) were used to evaluate the extent of genetic diversity within 23 rice genotypes which includes high yielding rice cultivars of *indica*, *aus*, *japonica*, aromatic rice and wild accessions. Among the 165 markers, 38 SSRs were monomorphic, and 50 SSRs were not considered for analysis because of ambiguous amplification. 77 (46.6 %) clearly amplified polymorphic SSRs distributed on all chromosomes were selected for the study (ESM_1). These 77 SSRs showed a total of 253 alleles at polymorphic loci. All the polymorphic loci revealed PIC values between 0.31 (RM12923) and 0.97 (RM3805), and more than 56 % loci showed PIC values in the range of 0.8–0.97. Allelic richness per locus varied from 2 (RM6464) to 9 (RM3805) with an average of 3.3 alleles. The allelic richness observed was compared to reports of Singh et al. (2013); Pachauri et al. (2013); Singh et al. (2014); Lang et al. (2014); however, it was lower when equated with Ram et al. (2007); Herrera et al. (2008); Upadhyay et al. (2012); Wang et al. (2013). The high polymorphism in these studies may be contributed by the number and diversity of germplasm accessions used including wild species and land races, and also by number and type of SSRs utilized in genotyping.

The SSR locus RM3805 on chromosome 6 showed 9 alleles in the 23 genotypes and had the highest PIC value of 0.97, followed by RM6842 on chromosome 2 with 8 alleles, and RM23914, RM7075, RM7485, RM3850, RM1369 with 6 alleles each on chromosomes 1, 2, 6 and 8, respectively. In the amplicons, di-nucleotide repeats (54.5 %) were found to be polymorphic, exhibiting high PIC values (>0.7) followed by tri-nucleotide and tetra-nucleotide repeats. In this study, an average PIC value of 0.79 was observed which is higher than reported in previous studies of Ravi et al. (2003) (0.57); Ram et al. (2007) (0.7); Upadhyay et al. 2012 (0.52); Pervaiz et al. (2010) (0.56); Singh et al. (2013) (0.25); Pachauri et al. (2013) (0.38); Babu et al. (2014) (0.50) and Singh et al. (2014) (0.42). This indicates that the SSR markers selected in this study were highly informative. Thus, the subset of rice universal core markers used in the study are useful for diversity analysis of a wide range of genotypes, especially between the cultivars and wild accessions because of their highly polymorphic nature and uniform distribution across the genome (Orjuela et al. 2010; Ali et al. 2010). The results will also be useful in selecting diverse genotypes and polymorphic SSR markers in inter-specific breeding programs and QTL mapping studies in rice. The polymorphic markers appear to be adequate in delineating accessions according to their lineage.

### Genotype specific alleles

Rare alleles (alleles with a frequency less than 5 %) were identified at 11 % loci, while the frequency of the most common allele at each locus ranged from 14 % (RM5748) to 95 % (RM295). Among the markers used, 23 SSRs amplified 28 unique genotype specific alleles (Table 2). The highest number of specific alleles were amplified by 9 SSRs in *O. rufipogon* (Acc No CR100309) followed by Nipponbare and *O. nivara* (Acc No CR100008). RM1018 on chromosome 4, RM8009 on chromosome 7 and RM273 on chromosome 12 amplified a unique allele each in the wild accessions. RM8131 on chromosome 1 was able to delineate wild accessions from *O. sativa* cultivars except N22, an *aus* type of *O. sativa* cultivar. RM425 could help differentiate *O. rufipogon* (Acc No CR100309) and *O.nivara* (Acc No CR100008) accessions clearly. RM8004 on chromosomal and RM5543 on chromosome 7 could help distinguish the 2 *O. rufipogon* accessions from the 2 *O. nivara* accessions. The popular rice variety MTU1010 was uniquely identified using RM7075, Swarna with RM3805 and Pusa Basmati1 with RM5752.

**Table 2:** SSR markers showing genotype-specific alleles

Genotypes	SSR markers showing unique alleles	Chr. no.
*O. rufipogon* (A) (Acc No: CR100267)	RM426	3
	RM6464	1
*O. rufipogon* (B) (Acc No: CR100309)	RM6775	6
	RM426	3
	RM5626	3
	RM5474	3
	RM3850	2
	RM6	2
	RM425	1
	RM140	1
	RM3362	1
*O. nivara* (C) (Acc No: CR100008)	RM1369	6
	RM7485	2
	RM425	2
*O. nivara* (D) (Acc No: CR100097)	RM3850	2
Nipponbare	RM7642	3
	RM3585	3
	RM507	5
	RM3372	3
	RM5474	3
	RM425	2
N22	RM8009	7
	RM6367	2
	RM23914	9
IR64	RM234	7
PusaBasmati-1	RM5752	7
Swarna	RM3805	6
MTU1010	RM7075	1

Among the SSR markers used in this study, three could distinguish wild accessions from indica and japonica types, and markers were identified which shows different alleles in the premium quality rices viz., basmati and BPT5204 from other genotypes under study, so that we can clearly distinguish any mixtures or adulteration at the commercial level. Nagaraju et al. (2002) reported SSR and ISSR marker assays which help in the determination of adulteration in basmati seed with non basmati seed. Clear distinction in the wild, *indica*, *japonica* and basmati types shows their independent evolution and the power of the core SSR markers and chloroplast SNPs to discriminate the genetic relationships Garris et al. (2005). Travis et al. (2015) studied genetic diversity among 511 cultivars from Bangladesh and North East India using a 384-SNP microarray assay. They identified 191, 229 and 142 SNPs clearly distinguish *indica*, *japonica* and *aus* accessions, respectively, and the *aus* group has been further resolved into two subpopulations *aus*1 and *aus*2.

### Conserved regions in the diverse germplasm

There were 38 SSR loci which were identified to be monomorphic or conserved in all the genotypes, and were distributed on all chromosomes except chromosome 7. Most of the monomorphic SSR markers showed unique single allele in all the genotypes. A few others showed either double bands (RM550, RM28118 and RM103) or multiple bands (RM6327, and RM6404) yet conserved in all the genotypes. Two SSR loci RM3805 and RM6314 showed double bands in most of the cultivars, but not in the wild accessions (ESM_2) indicating a locus duplication event during the domestication of rice.

It is interesting to note that many of these conserved monomorphic loci are reported to be associated with traits such as leaf senescence, seed dormancy, plant height, panicle number, pericarp color (Temnykh et al. 2000, 2001), and other yield related traits which are associated with crop domestication (ESM_3). It is possible that some of these monomorphic loci are linked with some important biological traits, but any major variation in these loci is probably not viable or not tolerated. Evidently, some of these loci are associated with fundamental developmental or morphological traits such as survival, adaptation, flowering and seed set.

### UPGMA cluster analysis

The dendrogram generated using UPGMA cluster analysis grouped 23 genotypes into three major clusters with Jacquard’s similarity coefficient ranging from 0.26 to 0.75 (ESM_4). The analysis revealed wide genetic variability among the genotypes. Cluster I consisted of wild accessions with genetic similarity ranging from 30 to 50 % which indicates their diversity in the rice gene pool. Similar findings of separation of wild accessions from cultivars were reported by Ravi et al. (2003) using 38 SSRs for evaluating 40 cultivars and 5 wild accessions, and Ram et al. (2007) using 25 SSRs to evaluate 35 genotypes. Cluster II was further divided into 3 sub clusters (A, B and C). Sub cluster-A, included popular high yielding genotypes viz, Swarna, MTU1010 and MTU1081 with a genetic similarity of more than 60 %. Sub cluster-B consisted of genotypes released from Indian Institute of Rice Research (IIRR) viz, Tulasi, Rasi, Dhanarasi, Varadhan and Krishnahamsa with a genetic similarity ranging from 49 to 53 %. Sub cluster-C comprised of BPT5204, IR64, Jaya and Moroberekan with a similarity coefficient from 0.42 to 0.68. It is noteworthy that in cluster II, the grouping of genotypes was associated with geographical origin or their center of development. High yielding Indian varieties viz, Swarna (MTU7029), MTU1010 and MTU1081 developed at Agricultural Research Station, Maruteru, Andhra Pradesh, grouped in sub cluster-A indicating their close genetic similarity or similarity of the parents in their pedigree. Sub cluster-B was composed of genotypes such as Rasi, Tulasi, Dhanarasi, Varadhan and Krishnahamsa developed at IIRR with two exceptions, Tellahamsa and Sahbhagidhan developed elsewhere. Sub cluster-C comprised of genotypes from different geographical origin which includes IR64 from IRRI, Philippines, and Jaya from IIRR, BPT5204 from Bapatla, Andhra Pradesh and Moroberekan, a tropical *japonica* type. This is also an indication that modern breeding programs involved development of highly suitable genotypes adapted to their local environments or quality preferences by crossing only the popular varieties for important agronomic traits (Babu et al. 2014). This emphasizes the need of widening the gene pool in rice by introgression of new genes from diverse sources (Ali et al. 2010). A close association among the genotypes in particular clusters is largely due to common pedigree involved in breeding of the genotypes. For instance, sub cluster-B of cluster II consists of genotypes Rasi, Tellahamsa, Jaya and BPT5204 which have TN1 as one common parent in their pedigree. Also, Tulasi and Krishnahamsa both derived from Rasi were grouped in sub cluster B. All these instances of close grouping support the robustness of the grouping even though it is based on 77 loci. Early maturing varieties viz, Rasi and Tulasi with a common pedigree in their breeding grouped in a sub cluster with a similarity index of more than 67 % (Ravidra Babu et al. 2015). Similarly the study on genetic diversity of Venezuelan cultivars by Herrera et al. 2008 and diversity of Brazilian rice cultivars by Raimondi et al. 2014, demonstrated the narrow genetic base of the cultivars developed in their respective countries which can lead to genetic vulnerability to any emerging stress conditions.

**Fig. 1:**
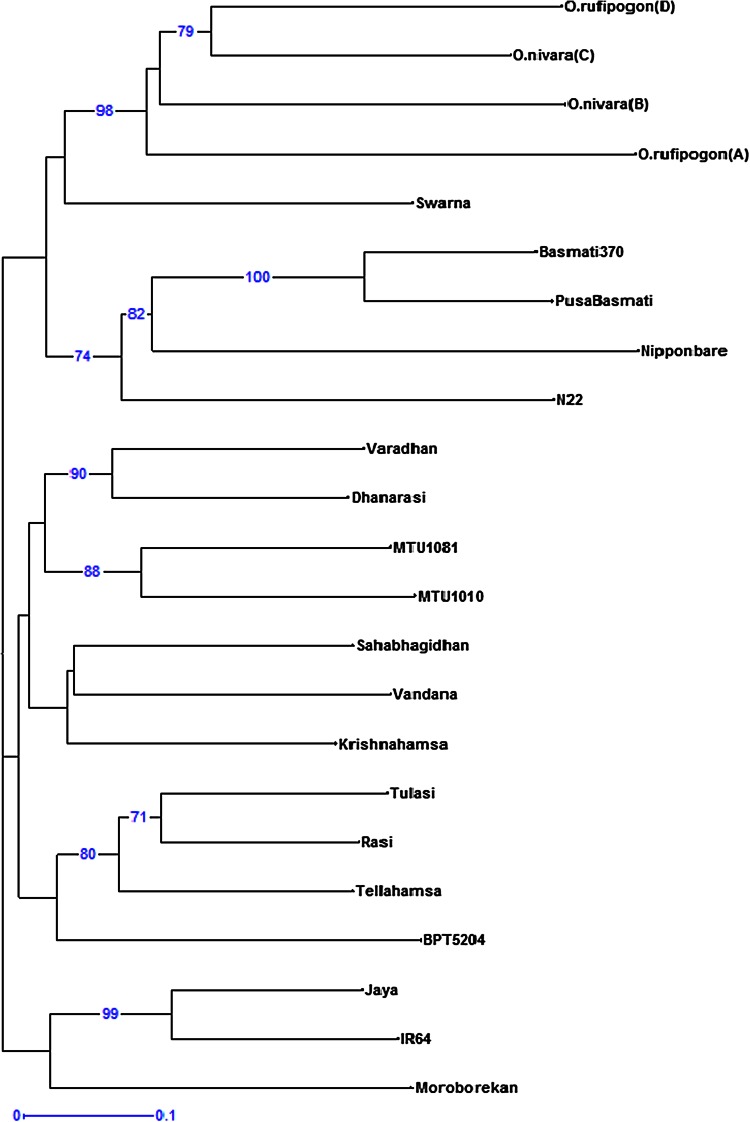
Neighbor joining tree illustrating the genetic relationships of the 23 genotypes

Cluster III included two aromatic rice genotypes Basmati370 and Pusa Basmati1 which are closely related with a genetic similarity of 75 %. The unrooted neighbor-joining tree (UNJ) constructed using DARwin was in agreement with the UPGMA analysis, where the genotypes grouped into three major clusters. The boot strap analysis was done for 100 permutations and the boot strap values above 50 were considered and depicted in Fig. 1. Most of the clusters showed moderate to high boot support (57–100 %).

The aromatic rice Pusa Basmati-1 is derived from Basmati370 as one of the parent and both showed high genetic similarity of 75 % between them and grouped in Cluster III. This largely confirms the accuracy of the grouping using the 77 core SSR markers in our study. The *japonica* genotypes Nipponbare, Moroberekan and *aus* type N22 grouped with *indica* genotypes Jaya, IR64, Pusa Basmati-1 and Basamti370. This grouping of indica- japonica- aus- aromatic might be due to an introgression of alleles from different sources during the course of evolution. The grouping of *japonica* genotype Moroberekan with *indica* type Tellahamsa and of *aus* type N22 with Basmati370 was reported previously as well (Chakravarthy and Ram Babu 2006; Upadhyay et al. 2012). Aromatic and *indica* genotypes grouped separately as also reported by Jain et al. (2004); Garris et al. (2005).

### Population structure

**Fig. 2:**
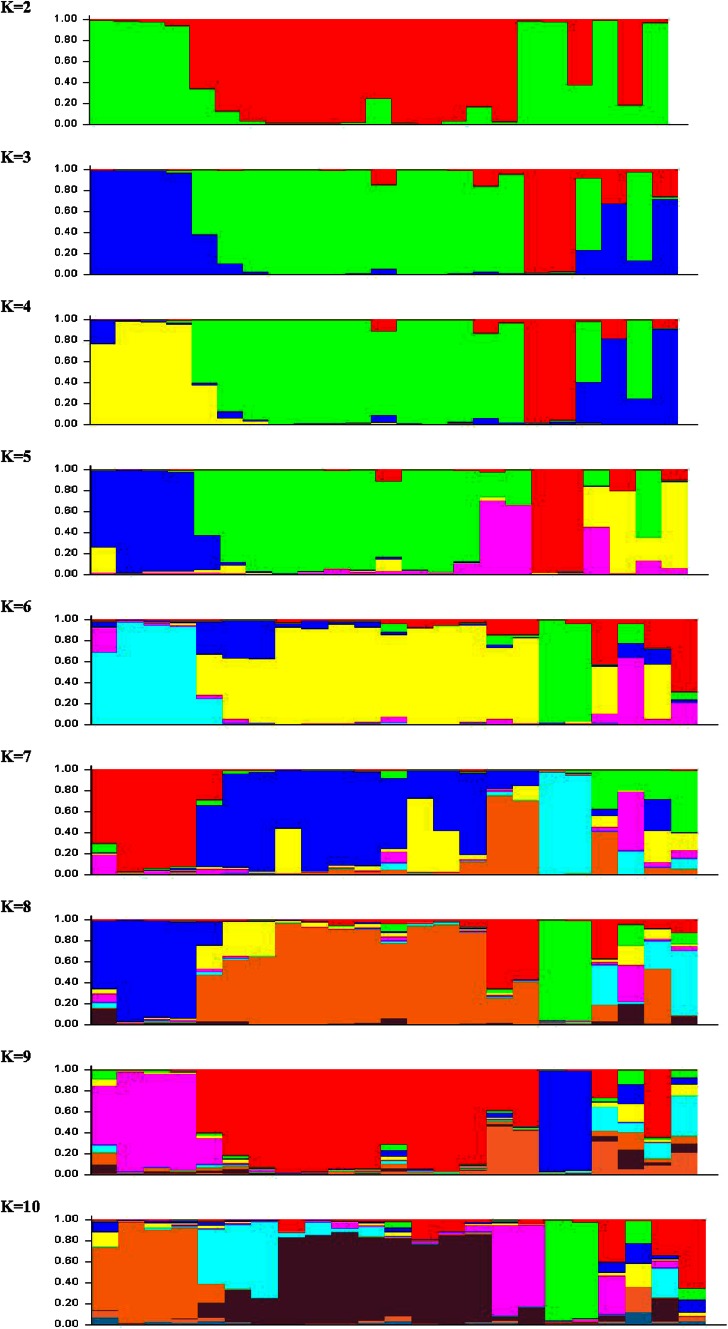
Population structure (STRUCTURE) at *K* = 2 to *K* = 10 of 23 rice genotypes based on genotypic data using 77 SSRs

Model-based program STRUCTURE 2.3.4 was used to determine the genetic relationship among individual rice accessions. Simulations were conducted based on admixture model with *K* range from 2 to 10 with 10 iterations using all 23 genotypes which showed significant population structure at *K* = 4 (Fig. 2). By comparing the LnP(D) and Evanno’s DK values by increasing *K* from 2 to 10, we found that LnP(D) values increased up to *K* = 4, with the highest log likelihood score at the same position. The population structure using SSRs showed that likelihood reached a sharp peak when the number of populations was set at four, suggesting that these rice accessions can be grouped into four subpopulations.

Populations were studied for the number of pure and admix individuals. At *K* = 4; population 1 had all wild accessions with 3 pure and 1 admix genotype, population 2 had 13 Indian cultivars with four admixes, population 3 consisted of popular cultivars Jaya and Nagina 22 and aromatic genotypes. In population 4, *japonica* cultivars were present with admixes from *indica* genotypes and many subgroups appeared within the major groups when *K* was increased from 4. The population grouping through structure analysis also showed similar result as that of the distance-based clustering. Studies on genetic diversity and population structure reported that domestication, geographical location and breeding objectives influenced the genetic structure of rice genotypes significantly (Garris et al. 2005; Lu et al. 2005; Yamamto et al. 2010; Chakhonkaen et al. 2012; Courtois et al. 2012; Yonemaru et al. 2012; Choudhury et al. 2013; Shinada et al. 2014; Huang et al. 2015). Population structure results of this study are in confirmation with global classification of rice germplasm by Garris et al. (2005), Wang et al. (2014) into *indica*, *aus*, aromatic, temperate *japonica* and tropical *japonica*. Wild accessions of *O. nivara* and *O. rufipogon* showed a clear differentiation from cultivated germplasm.

### Principal component analysis

**Fig. 3:**
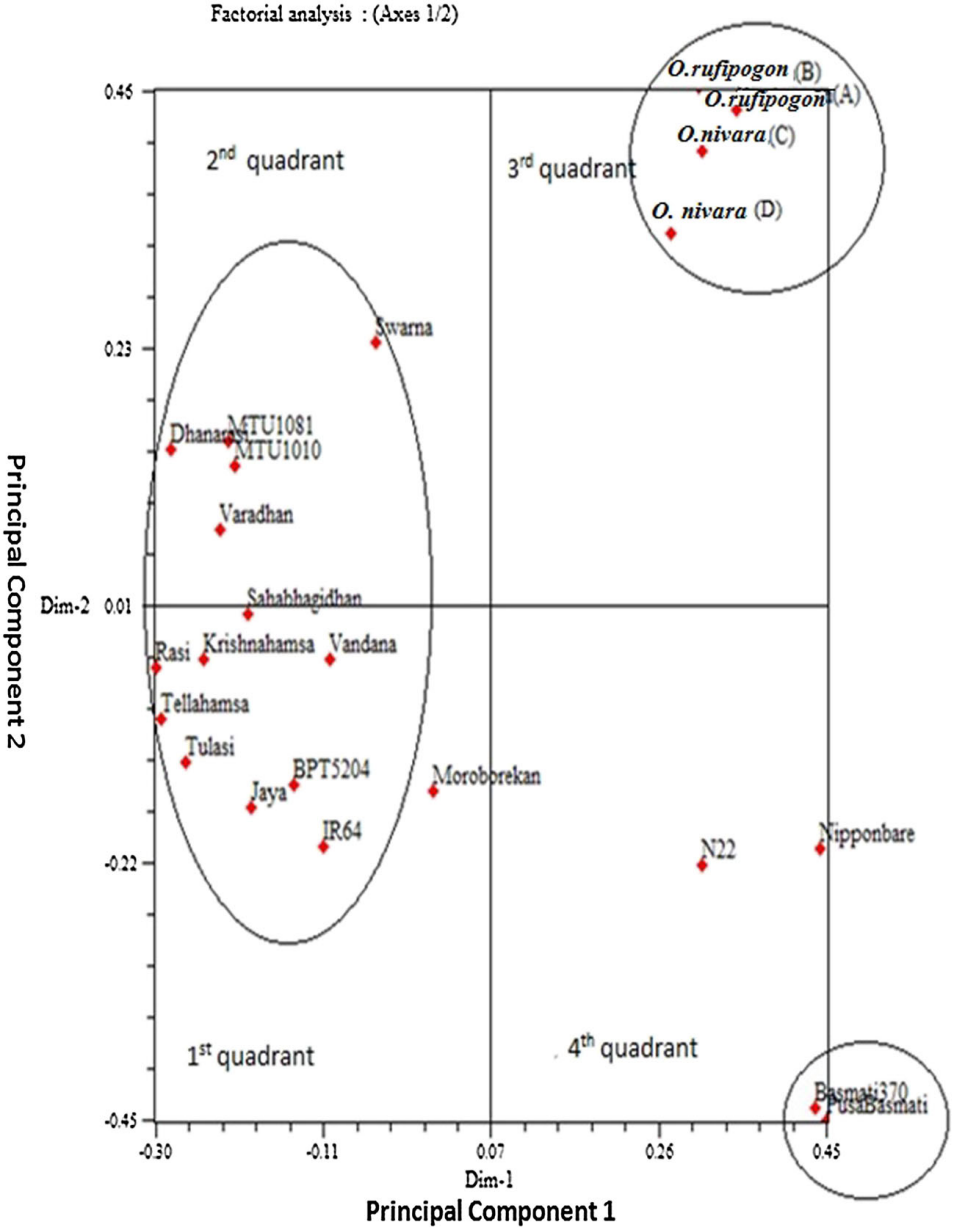
Two-dimensional scaling of principal component analysis of 23 genotypes based on genotypic data using 77 SSRs

Principal component analysis (PCA) was conducted to understand the genetic relationships among the elite breeding lines and with the wild accessions. Among the 23 genotypes, 4 wild accessions formed a distinct cluster in the upper-right corner of 3rd quadrant, showing a clear distinction from cultivars in the two-dimensional dispersion of all genotypes (Fig. 3). The analysis for genotypes separated the accessions into four clearly separated groups with two basmati lines positioned in the bottom right corner of the diagram in the fourth quadrant, and the *japonica* genotype, Nipponbare and *aus* genotype. N22 were located close to the basmati genotypes in the same quadrant. All the Indian popular rice varieties were present in the left half of the diagram mainly in 1st and 2nd quadrant along with Moroberekan, a tropical *japonica* genotype.

The first three principal components had Eigen values of 44.96, 10.32 and 9.46 %, and an overall maximum cumulative variation of 65 % were observed with first three components of principal coordinates. Two-dimensional scaling obtained using PCA analysis also showed the same grouping pattern as dendrogram and sorted most of the cultivars into three major clusters distributed across the quadrants. This technique has been used to partition rice genotypes based on variation in molecular as well as morphological data. Similar pattern of Eigen values were observed for first principal components in classifying rice subgroups by Seetharam et al. (2009); Maji and Shaibu (2012); Gana et al. (2013); Nachimuthu et al. (2015). Since the variation is high (≥25 %) for principal components, this information can be used along with cluster analysis to identify the related genotypes (Ahmad et al. 2015). Our results are in agreement with Adebisi et al. (2012), who reported that the first three principal components were the most important in reflecting the variation patterns among accessions. The markers associated in classifying the genotypes can be recommended in differentiating population subgroups. Comparison of PCA and dendrogram groupings revealed generally similar trends, with a minor difference in composition of clusters.

## Conclusion

The present study confirmed the potential of the 77 SSRs which is a subset of universal core set SSRs (Orjuela et al. 2010) in differentiating the wild accessions from other genotypes, and were highly polymorphic, informative and had uniform distribution across the genome. They are useful for diversity analysis; marker assisted breeding programs and QTL mapping studies in rice. Genotype specific alleles identified can be utilized as species-specific diagnostic markers to identify the rice varieties in mixtures or for adulteration check when the seed morphology shows no variation. Cultivar specific alleles generated by SSR markers would be useful in variety identification and germplasm characterization after reconfirming in large population. Genotypic grouping through structure analysis, distance-based clustering and principal component analysis were similar confirming the results. Most of the genotypes grouped according to the geographical region, pedigree and genetic similarity. The diverse chromosomal segments harboring unique alleles specific to wild accessions of *O. nivara* and *O. rufipogon* can be harnessed for the development of chromosome segment substitution lines, mapping and transferring valuable genes from wild to cultivated rice. Distant genotypes identified in the study can be used in hybridization programs for further improvement of rice germplasm to meet the future food grain requirements. Our analysis shows the close relationship of high yielding popular Indian varieties which share a similar gene pool as they originated from a few common parents. It underlines the concern that breeding within the popular cultivars will further narrow down the genetic base of an available gene pool. Breeding activities will benefit if focus is more on crosses between distant genotypes or to enhance the diversity and identification of new genes and QTLs from untapped resources for further crop improvement.

## Electronic supplementary material

Below is the link to the electronic supplementary material.

**ESM_1**: List of polymorphic markers used. **ESM _2**: Conserved and polymorphic loci identified in the genotypes. **ESM _3**: List of monomorphic primers identified in diversity study and the associated traits. **ESM _4**: Dendrogram based on genotypic data for 253 alleles at 77 SSR loci in 23 rice genotypes showing three major groups (DOC 1496 kb)
