# Supplementary material for: Genetic characterization and population structure of Indian rice cultivars and wild genotypes using core set markers
Source: 3 Biotech. 2016 Mar 26;6(1):95. doi: 10.1007/s13205-016-0409-7 (PMC4808523; doi:10.1007/s13205-016-0409-7)
Supplement: Supplementary file 1 — ESM_1: List of polymorphic markers used. ESM _2: Conserved and polymorphic loci identified in the genotypes. ESM _3: List of monomorphic primers identified in diversity study and the associated traits. ESM _4: Dendrogram based on genotypic data for 253 alleles at 77 SSR loci in 23 rice genotypes showing three major groups (DOC 1496 kb) [file 13205_2016_409_MOESM1_ESM.doc]

Title: Genetic characterization and population structure of Indian rice cultivars and wild genotypes using core set markers

Journal : 3Biotech

Malathi Surapaneni, Divya Balakrishnan*, Sukumar Mesapogu, Addanki Krishnamraju, Yadavalli Venkateswara Rao and Sarla Neelamraju

ICAR-National Professor Project, Directorate of Rice Research, Hyderabad, India

Corresponding author : [*divyab0005@gmail.com**](mailto:divyab0005@gmail.com*)

**ESM _1**: List of polymorphic markers used in the study

| **S. No** | **Locus** | **Primer Sequence (5'-3')** | **Chr No** | **SSR Motif** | **Size** | **No of Alleles** | **PIC** |
| --- | --- | --- | --- | --- | --- | --- | --- |
|  | RM6464 | F: ACACTCTCTCTCCTCGCTGC | 1 | (GCC)8 | 146 | 2 | 0.53 |
|  |  | R: CGAGGAGAATACTCGTTCGG |  |  |  |  |  |
|  | RM6470 | F: ACCTTTCCCATGGTGGAATC | 1 | (GCC)8 | 175 | 2 | 0.7 |
|  |  | R: TTATACCTTCGACGGGAACG |  |  |  |  |  |
|  | RM1329 | F: GAGCTCAATCGAATCTAGACC | 1 | RM1329 | 151 | 3 | 0.88 |
|  |  | R: ATTGACATTCCTTTGCTTTG |  |  |  |  |  |
|  | RM8131 | F: ACCGGCGGTCCTAATAACTATGG | 1 | RM8131 | 139 | 5 | 0.79 |
|  |  | R: GGTCCGATTAGATGAGAATCAGTTGG |  |  |  |  |  |
|  | RM140 | F: TGCCTCTTCCCTGGCTCCCCTG | 1 | (CT)12 | 261 | 3 | 0.82 |
|  |  | R: GGCATGCCGAATGAAATGCATG |  |  |  |  |  |
|  | RM7075 | F: TATGGACTGGAGCAAACCTC | 1 | (ACAT)13 | 155 | 6 | 0.95 |
|  |  | R: GGCACAGCACCAATGTCTC |  |  |  |  |  |
|  | RM8004 | F: TTGACCAAAGGTGATTGTAAT | 1 | (TCA)18 | 149 | 4 | 0.92 |
|  |  | R: CTTGATGAGTTTCATGAGCA |  |  |  |  |  |
|  | RM11307 | F: AAAGCTCTGCAATCTTCTCTCC | 1 | (GA)15 | 147 | 3 | 0.85 |
|  |  | R: GAATACGACATCAGAACAGTGC |  |  |  |  |  |
|  | RM5794 | F: AGCTAGCTGAGCTCGTCGTC | 1 | (AGC)8 | 151 | 2 | 0.73 |
|  |  | R: CAGACTCATGGACACATGGG |  |  |  |  |  |
|  | RM3362 | F: AAGTTGAAGCAGTCGCCAAC | 1 | (CT)15 | 139 | 3 | 0.74 |
|  |  | R: GAATTGCGTGGGATATGGAC |  |  |  |  |  |
|  | RM6842 | F: TAAATCGAAGGAGGGGGAAG | 2 | (TCT)21 | 196 | 8 | 0.64 |
|  |  | R: GGAAGAAGGAGGAGGAGGTG |  |  |  |  |  |
|  | RM6367 | F: CAGACAGAACAGCGGTCAAG | 2 | (GAA)14 | 202 | 5 | 0.94 |
|  |  | R: GATGGATGGATGGATTGGAG |  |  |  |  |  |
|  | RM4355 | F: GGGATGAGAGTAGAAGGCA | 2 | (TA)18 | 142 | 3 | 0.8 |
|  |  | R: TATATGGCAAGCCTAGCG |  |  |  |  |  |
|  | RM12729 | F: TGGAGCAGCTCAAGATAGTAGACC | 2 | (AT)33 | 199 | 2 | 0.79 |
|  |  | R: TATTTGGATTCCGTTCCAGACG |  |  |  |  |  |
|  | RM12923 | F: AAATGCACAGGCATTCGTAGACC | 2 | (CCT)7 | 216 | 2 | 0.31 |
|  |  | R: GAAGAAGTGGATGGAGGACATGG |  |  |  |  |  |
|  | RM5430 | F: TAAAAACTGAGCCGTGAGCC | 2 | (TC)16 | 181 | 2 | 0.57 |
|  |  | R: ACCATGGGGAGCTGCTTC |  |  |  |  |  |
|  | RM6 | F: GTCCCCTCCACCCAATTC | 2 | (AG)16 | 163 | 4 | 0.93 |
|  |  | R: TCGTCTACTGTTGGCTGCAC |  |  |  |  |  |
|  | RM425 | F: CCAACGAAGATTCGAAGCTC | 2 | (CGG)9 | 126 | 4 | 0.82 |
|  |  | R: CAGCACCATGAAGTCGCC |  |  |  |  |  |
|  | RM3850 | F: AAGTTGAGAATGAGGGACAA | 2 | (GA)24 | 110 | 6 | 0.96 |
|  |  | R: TTCGGAAGTGAAAAGGTAAT |  |  |  |  |  |
|  | RM7485 | F: GCCAGTTTCTCCAAAAGACG | 2 | (TATC)7 | 161 | 6 | 0.94 |
|  |  | R: AACTAGCCTCGACAGCGAAC |  |  |  |  |  |
|  | RM14303 | F: GTCTGTGCGCTCCTTGTTCTAGC | 3 | (AC)19 | 191 | 3 | 0.88 |
|  |  | R: CCTGGACCAATTTGTATGGTTGG |  |  |  |  |  |
|  | RM3372 | F: GAGCGACCAAAGAATCCAAG | 3 | (CT)16 | 84 | 3 | 0.76 |
|  |  | R: CCACGGGGAGCTGATGAAG |  |  |  |  |  |
|  | RM5474 | F: AAAGTGTTGGTGAGCATAGC | 3 | (TC)21 | 155 | 5 | 0.9 |
|  |  | R: TTTGTGTTTGGAGAGACGAG |  |  |  |  |  |
|  | RM1256 | F: ACGCGAAGCAACGGAGATAG | 3 | (AG)16 | 148 | 3 | 0.86 |
|  |  | R: CTAGCCTCGATGCGAAAAAC |  |  |  |  |  |
|  | RM5748 | F: CAGTTGGCAATTGTCACGAG | 3 | (ACG)12 | 167 | 2 | 0.6 |
|  |  | R: TCGAACATATCCAAGCCTCC |  |  |  |  |  |
|  | RM7642 | F: ACGAAATATCAGGGCACCTG | 3 | (TTTA)7 | 194 | 3 | 0.71 |
|  |  | R: GTTGACTTTGGTCATGAGGG |  |  |  |  |  |
|  | RM3646 | F: ACTAGAGCACCCTCGCTGAG | 3 | (GA)14 | 137 | 3 | 0.9 |
|  |  | R: CTCAGCCACCCCATCAAC |  |  |  |  |  |
|  | RM5626 | F: ATCAGTCGGTCATAAACGCC | 3 | (AAG)11 | 188 | 5 | 0.95 |
|  |  | R: ACCTTCCTCTTCTGCTGCTG |  |  |  |  |  |
|  | RM426 | F: ATGAGATGAGTTCAAGGCCC | 3 | (CA)10 | 150 | 5 | 0.91 |
|  |  | R: AACTCTGTACCTCCATCGCC |  |  |  |  |  |
|  | RM422 | F: TTCAACCTGCATCCGCTC | 3 | (AG)30 | 385 | 2 | 0.61 |
|  |  | R: CCATCCAAATCAGCAACAGC |  |  |  |  |  |
|  | RM3585 | F: TTGATGAGAGTGAACCAGCG | 3 | (GA)12 | 169 | 2 | 0.65 |
|  |  | R: CGTTTATGCAGAACAACCCC |  |  |  |  |  |
|  | RM16368 | F: TGTCCAGAGAATGACAAAGTACGC | 4 | (TAAT)5 | 123 | 2 | 0.74 |
|  |  | R: GGATGTATATCTGCCACCAAATGC |  |  |  |  |  |
|  | RM16493 | F: TCGGCAGCAACAACTCTTAAACC | 4 | (CCT)7 | 174 | 2 | 0.74 |
|  |  | R: TCAACAAGATGACTCCCTGTAGCC |  |  |  |  |  |
|  | RM16559 | F: CCTGGAACCTGGAGGTGTTCTCG | 4 | (CCG)7 | 197 | 2 | 0.6 |
|  |  | R: GTCGTGGACGATTTCTTCGTCAGC |  |  |  |  |  |
|  | RM6314 | F: GATTCGTGTCGGTTGTCAAG | 4 | (CTT)11 | 169 | 2 | 0.37 |
|  |  | R: GGTTCAGGGACGAATTTCAG |  |  |  |  |  |
|  | RM273 | F: GAAGCCGTCGTGAAGTTACC | 4 | (GA)11 | 207 | 4 | 0.93 |
|  |  | R: GTTTCCTACCTGATCGCGAC |  |  |  |  |  |
|  | RM1018 | F: ATCTTGTCCCACTGCACCAC | 4 | (AC)13 | 160 | 3 | 0.86 |
|  |  | R: TGTGACTGCTTTTCTGTCGC |  |  |  |  |  |
|  | RM17377 | F: ATATTACTTCGACGCTGGATCAGG | 4 | (AG)25 | 168 | 4 | 0.92 |
|  |  | R: GTCAGTTCGTCAGGCACAACG |  |  |  |  |  |
|  | RM349 | F: TTGCCATTCGCGTGGAGGCG | 4 | (GA)16 | 136 | 2 | 0.76 |
|  |  | R: GTCCATCATCCCTATGGTCG |  |  |  |  |  |
|  | RM17604 | F: TCCTCTCCTCTCCGATCCTTAGC | 4 | (AG)10 | 149 | 2 | 0.68 |
|  |  | R: CACATCACAACCACAAACCATGC |  |  |  |  |  |
|  | RM559 | F: ACGTACACTTGGCCCTATGC | 4 | (AACA)6 | 160 | 3 | 0.82 |
|  |  | R: ATGGGTGTCAGTTTGCTTCC |  |  |  |  |  |
|  | RM507 | F: CTTAAGCTCCAGCCGAAATG | 5 | (AAGA)7 | 258 | 3 | 0.8 |
|  |  | R: CTCACCCTCATCATCGCC |  |  |  |  |  |
|  | RM1024 | F: GCATATACCATGGGGATTGG | 5 | (AC)13 | 141 | 3 | 0.87 |
|  |  | R: GGGATTGGGATAATGGTGTG |  |  |  |  |  |
|  | RM17959 | F: AGAGATGGAAGTCTGGTGCTTTGG | 5 | (GA)14 | 268 | 2 | 0.49 |
|  |  | R: TCAACATAGCTTCAGGGCACCTAGC |  |  |  |  |  |
|  | RM18614 | F: TGGCGCAATATCTCTCTCATTCC | 5 | (TC)12 | 163 | 2 | 0.75 |
|  |  | R: TGCCACTTGTGTGTTGTTCTGC |  |  |  |  |  |
|  | RM3664 | F: CGCCTGCAAAAAAGGTAGAG | 5 | (GA)14 | 136 | 3 | 0.85 |
|  |  | R: GATCAAAGGAACCCCCGTAG |  |  |  |  |  |
|  | RM5907 | F: TGCTGTCTCCACTTCCCTTC | 5 | (ATT)19 | 187 | 3 | 0.88 |
|  |  | R: AAGGAGGCGTGCTTAACAGG |  |  |  |  |  |
|  | RM6775 | F: GCAGATCAAGTATGCCTGCC | 6 | (TCC)8 | 192 | 3 | 0.76 |
|  |  | R: TCGCTAGATAGGGGATGTGG |  |  |  |  |  |
|  | RM1369 | F: AACCTGAGAGTGCCAATTGG | 6 | (AG)27 | 125 | 6 | 0.95 |
|  |  | R: TCCCCTAGTAAAGCGGATTC |  |  |  |  |  |
|  | RM3805 | F: AGAGGAAGAAGCCAAGGAGG | 6 | (GA)19 | 110 | 9 | 0.97 |
|  |  | R: CATCAACGTACCAACCATGG |  |  |  |  |  |
|  | RM19620 | F: GCGACGAGGAAGAAGATTAGTTCG | 6 | (GTG)7 | 167 | 4 | 0.85 |
|  |  | R: GCGGCACTTCGAGCAGTACG |  |  |  |  |  |
|  | RM3431 | F: CCAATGGTGCAGTTAACGTG | 6 | (CT)18 | 161 | 3 | 0.83 |
|  |  | R: AACATTCTGGAAGACACGGG |  |  |  |  |  |
|  | RM3183 | F: GCTCCACAGAAAAGCAAAGC | 6 | (CT)12 | 140 | 2 | 0.67 |
|  |  | R: TGCAACAGTAGCTGTAGCCG |  |  |  |  |  |
|  | RM20069 | F: GCGAGCGAGAGGAGAGATAGACG | 6 | (CAGG)5 | 157 | 4 | 0.9 |
|  |  | R: CGAATTCGGCACGAGTAATAGGG |  |  |  |  |  |
|  | RM295 | F: CGAGACGAGCATCGGATAAG | 7 | (GA)2A(GA)3G2(GA)9 | 180 | 3 | 0.69 |
|  |  | R: GATCTGGTGGAGGGGAGG |  |  |  |  |  |
|  | RM20882 | F: CCATTTCATACCTCCATCCATCG | 7 | (AG)10 | 266 | 2 | 0.73 |
|  |  | R: ATTTGCAAACTAGGTCGGTCAGC |  |  |  |  |  |
|  | RM5752 | F: TTGCAATTAATTCGATCTCC | 7 | (ACT)13 | 138 | 3 | 0.88 |
|  |  | R: GCAGATCGATTCGTTAGTTC |  |  |  |  |  |
|  | RM8009 | F: TGTGACTCATCCTAATACAA | 7 | (AT)19 | 120 | 5 | 0.93 |
|  |  | R: ATCTGGATATATCTGTCCAA |  |  |  |  |  |
|  | RM21238 | F: GAGCTTCTCCTCACCCATCACC | 7 | (CGG)10 | 188 | 3 | 0.73 |
|  |  | R: CTTCTGCAGAGGGTGTTCAACG |  |  |  |  |  |
|  | RM5543 | F: ACCACTTGCTGGAATCCTTG | 7 | (TG)14 | 167 | 3 | 0.88 |
|  |  | R: GCAAATTCTGGGCTATCTGC |  |  |  |  |  |
|  | RM11 | F: TCTCCTCTTCCCCCGATC | 7 | (GA)17 | 140 | 2 | 0.76 |
|  |  | R: ATAGCGGGCGAGGCTTAG |  |  |  |  |  |
|  | RM6403 | F: GAACACGCCAAGAAACCAACC | 7 | (GAG)8 | 98 | 2 | 0.74 |
|  |  | R: TAGCACTGCGTAGCAGCAATGG |  |  |  |  |  |
|  | RM234 | F: ACAGTATCCAAGGCCCTGG | 7 | (CT)25 | 156 | 3 | 0.78 |
|  |  | R: CACGTGAGACAAAGACGGAG |  |  |  |  |  |
|  | RM118 | F: CCAATCGGAGCCACCGGAGAGC | 7 | (GA)8 | 156 | 2 | 0.72 |
|  |  | R: CACATCCTCCAGCGACGCCGAG |  |  |  |  |  |
|  | RM420 | F: GGACAGAATGTGAAGACAGTCG | 7 | (AAAT)7 | 197 | 2 | 0.61 |
|  |  | R: ACTAATCCACCAACGCATCC |  |  |  |  |  |
|  | RM408 | F: CAACGAGCTAACTTCCGTCC | 8 | (CT)13 | 128 | 3 | 0.86 |
|  |  | R: ACTGCTACTTGGGTAGCTGACC |  |  |  |  |  |
|  | RM1376 | F: CATGTGTGATGACTGACAGG | 8 | (AG)31 | 199 | 4 | 0.92 |
|  |  | R: GGTGCTGTGATGATTCTTTC |  |  |  |  |  |
|  | RM447 | F: CCCTTGTGCTGTCTCCTCTC | 8 | (CTT)8 | 111 | 3 | 0.92 |
|  |  | R: ACGGGCTTCTTCTCCTTCTC |  |  |  |  |  |
|  | RM23914 | F: GAGGATCCTTACCATCAAACTTCG | 9 | (AT)23 | 196 | 6 | 0.96 |
|  |  | R: CCAAGAACCTGCATTCTTCAAGG |  |  |  |  |  |
|  | RM296 | F: CACATGGCACCAACCTCC | 9 | (GA)10 | 123 | 3 | 0.84 |
|  |  | R: GCCAAGTCATTCACTACTCTGG |  |  |  |  |  |
|  | RM3249 | F: GCCCTTTTCTTCTCCACTCC | 9 | (CT)13 | 151 | 3 | 0.85 |
|  |  | R: AGACACTGTCACAGCTTCAGC |  |  |  |  |  |
|  | RM1026 | F: GCCTCTGGCAGAATAGCATC | 9 | (AC)14 | 164 | 3 | 0.85 |
|  |  | R: TATCACTTTGCTGCCTAGGC |  |  |  |  |  |
|  | RM239 | F: TACAAAATGCTGGGTACCCC | 10 | (AG)5TG(AG)2 | 144 | 3 | 0.78 |
|  |  | R: ACATATGGGACCCACCTGTC |  |  |  |  |  |
|  | RM6673 | F: CATCGCATCGTATCGTATCG | 10 | (TAA)10 | 147 | 3 | 0.84 |
|  |  | R: GCTTCAAACACGCCTTCTTC |  |  |  |  |  |
|  | RM26105 | F: AGCATCAATTCAGCTTGCTTGC | 11 | (TC)10 | 139 | 3 | 0.86 |
|  |  | R: TTCTGGTTCTTTGAGAGAGTGTGC |  |  |  |  |  |
|  | RM7315 | F: CACAAAGGCGTGTGGGTTAG | 12 | (CACG)9 | 189 | 3 | 0.86 |
|  |  | R: GAGTCACGGGATGTTGCC |  |  |  |  |  |
|  | RM1159 | F: GTTGATGGTGTCAGCGAGAG | 12 | (AG)13 | 169 | 2 | 0.69 |
|  |  | R: ACATTTGCACCACACCACAG |  |  |  |  |  |

| Title: Genetic characterization and population structure of Indian rice cultivars and wild genotypes using core set markers  Journal : 3Biotech  Malathi Surapaneni, Divya Balakrishnan*, Sukumar Mesapogu, Addanki Krishnamraju, Yadavalli Venkateswara Rao and Sarla Neelamraju  ICAR-National Professor Project, Directorate of Rice Research, Hyderabad, India  Corresponding author : [*divyab0005@gmail.com**](mailto:divyab0005@gmail.com*)  **ESM _2 : Conserved and polymorphic loci identified in the genotypes under study**  The order of 23genotypes in the gel picture was according to Table 1. |
| --- |
| **Monomorphic loci with single allele**  1 2 3 4 5 6 7 8 9 10 11 12 13 14 15 16 17 18 19 20 21 22 23 |
| 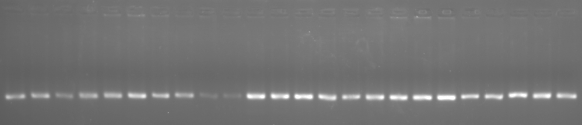 |
| RM16559  1 2 3 4 5 6 7 8 9 10 11 12 13 14 15 16 17 18 19 20 21 22 23 |
| 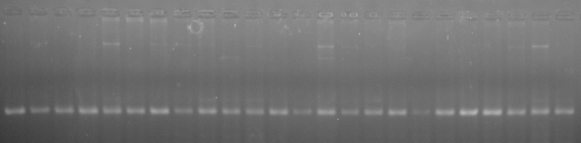 |
| 16140 |
| **Monomorphic loci with Double bands** |
| 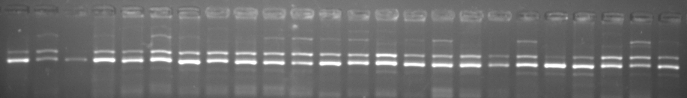  1 2 3 4 5 6 7 8 9 10 11 12 13 14 15 16 17 18 19 20 21 22 23 |
| RM550 |
| 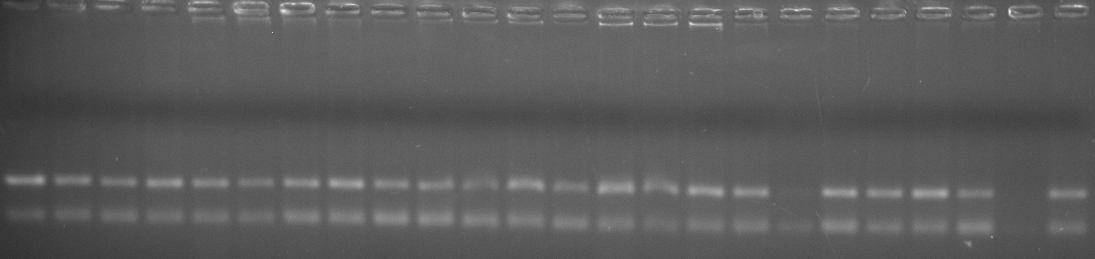  1 2 3 4 5 6 7 8 9 10 11 12 13 14 15 16 17 18 19 20 21 22 23 |
| RM28118 |
| **Monomorphic loci with multiple bands** |
| 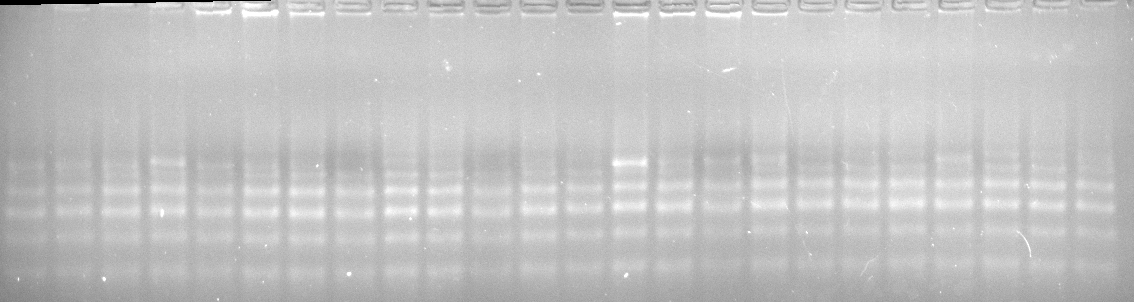  1 2 3 4 5 6 7 8 9 10 11 12 13 14 15 16 17 18 19 20 21 22 23 |
| RM6404 |
| 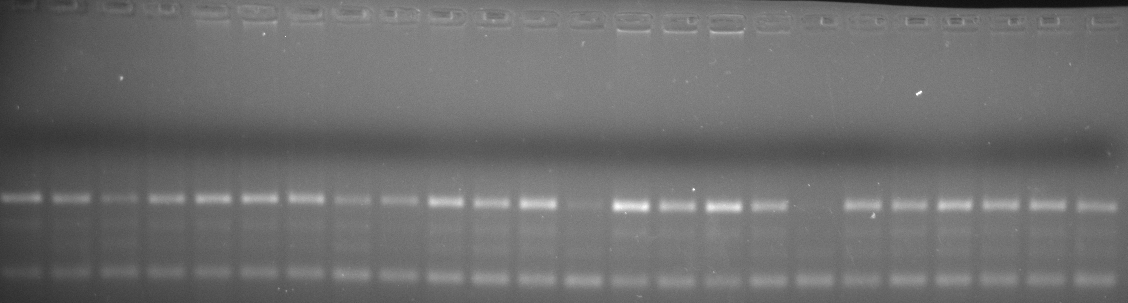  1 2 3 4 5 6 7 8 9 10 11 12 13 14 15 16 17 18 19 20 21 22 23 |
| RM25365 |

**Polymorphic SSR loci**


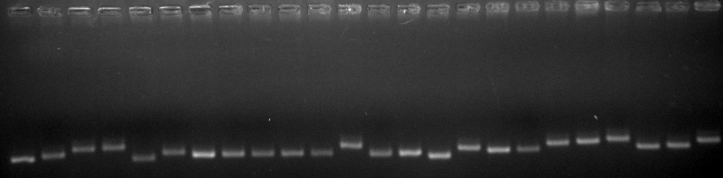


1 2 3 4 5 6 7 8 9 10 11 12 13 14 15 16 17 18 19 20 21 22 23

RM11307


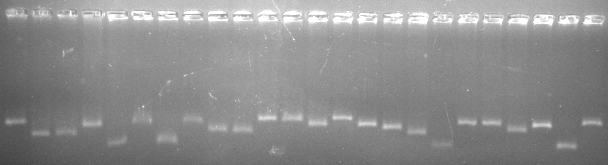


1 2 3 4 5 6 7 8 9 10 11 12 13 14 15 16 17 18 19 20 21 22 23

RM8004

| Title: Genetic characterization and population structure of Indian rice cultivars and wild genotypes using core set markers  Journal : 3Biotech  Malathi Surapaneni, Divya Balakrishnan*, Sukumar Mesapogu, Addanki Krishnamraju, Yadavalli Venkateswara Rao and Sarla Neelamraju  ICAR-National Professor Project, Directorate of Rice Research, Hyderabad, India  Corresponding author : *divyab0005@gmail.com**  **ESM _3: List of Monomorphic primers identified in diversity study and the traits associated** | | | |
| --- | --- | --- | --- |
|  | | | |
| **Sl. No.** | **SSR loci** | **Chr. No** | **Traits associated(http://www.gramene.org/)** |
| 1 | RM272 | 1 | Days to heading, grain number, spikelet number, panicle number, plant height, harvest index |
| 2 | RM11635 | 1 | NA |
| 3 | RM11893 | 1 | NA |
| 4 | RM12923 | 2 | NA |
| 5 | RM550 | 2 | leaf senescence, panicle number, chlorophyll content |
| 6 | RM5430 | 2 | NA |
| 7 | RM6617 | 2 | NA |
| 8 | RM6307 | 2 | NA |
| 9 | RM6509 | 2 | NA |
| 10 | RM221 | 2 | Root number, root thickness, BPH resistance, spikelet number, hot paste viscosity, breakdown viscosity, plant height, grain number, grain yield/plant, crushed grain %, head rice, Leaf senescence, panicle length, days to heading, seedling vigour, spikelet fertility, tiller angle, total shoot elongation under submergence, harvest index. |
| 11 | RM3654 | 3 | NA |
| 12 | RM14593 | 3 | NA |
| 13 | RM16140 | 3 | NA |
| 14 | RM142 | 4 | Rhizome number, rhizome branching angle and number, rhizome internode length, rhizome length, culm thickness, filled grain number, scent, chlorophyll content, tiller number, rhizome dry weight, leaf senescence, |
| 15 | RM16705 | 4 | NA |
| 16 | RM537 | 4 | seed weight |
| 17 | RM7588 | 5 | NA |
| 18 | RM19976 | 6 | NA |
| 19 | RM103 | 6 | consistency viscosity, gelatinization temperature, set back |
| 20 | RM22883 | 8 | NA |
| 21 | RM6193 | 8 | NA |
| 22 | RM149 | 8 | BPH resistance, plant height, days to heading, panicle length, spikelet fertility, leaf width, osmotic adjustment capacity, filled grain number, leaf senescence, grain number, spikelets per panicle length, grain yield per plant, seedling vigor, leaf width, panicle number, culm length, 1000 seed weight, scent, flour colour |
| 23 | RM477 | 8 | Tiller number, spikelet fertility, root thickness. |
| 24 | RM23654 | 9 | NA |
| 25 | RM23736 | 9 | NA |
| 26 | RM23769 | 9 | NA |
| 27 | RM409 | 9 | Tiller number, root length. |
| 28 | RM216 | 10 | Rhizome number, rhizome internode length, rhizome length, rhizome dry weight, BPH resistance, rhizome internode number, root length, spikelet fertility, internode length, grain yield per plant, days to heading, total shoot elongation under submergence, spikelet number,1000-seed weight, culm strength, plant survival percentage under submergence, submergence sensitivity, leaf senescence |
| 29 | RM25365 | 10 | NA |
| 30 | RM184 | 10 | Cold tolerance, leaf senescence, days to heading, |
| 31 | RM147 | 10 | Leaf senescence, seed dormancy, pericarp colour, panicle length, seed set percentage |
| 32 | RM25663 | 10 | NA |
| 33 | RM6327 | 11 | NA |
| 34 | RM27318 | 11 | NA |
| 35 | RM26796 | 11 | NA |
| 36 | RM26062 | 11 | NA |
| 37 | RM27706 | 12 | NA |
| 38 | RM28118 | 12 | NA |

| Title: Genetic characterization and population structure of Indian rice cultivars and wild genotypes using core set markers  Journal : 3Biotech  Malathi Surapaneni, Divya Balakrishnan*, Sukumar Mesapogu, Addanki Krishnamraju, Yadavalli Venkateswara Rao and Sarla Neelamraju  ICAR-National Professor Project, Directorate of Rice Research, Hyderabad, India  Corresponding author : [*divyab0005@gmail.com**](mailto:divyab0005@gmail.com*)  **ESM _4: Dendrogram based on genotypic data for 253 alleles at 77 SSR loci in 23 rice genotypes showing three major groups**  **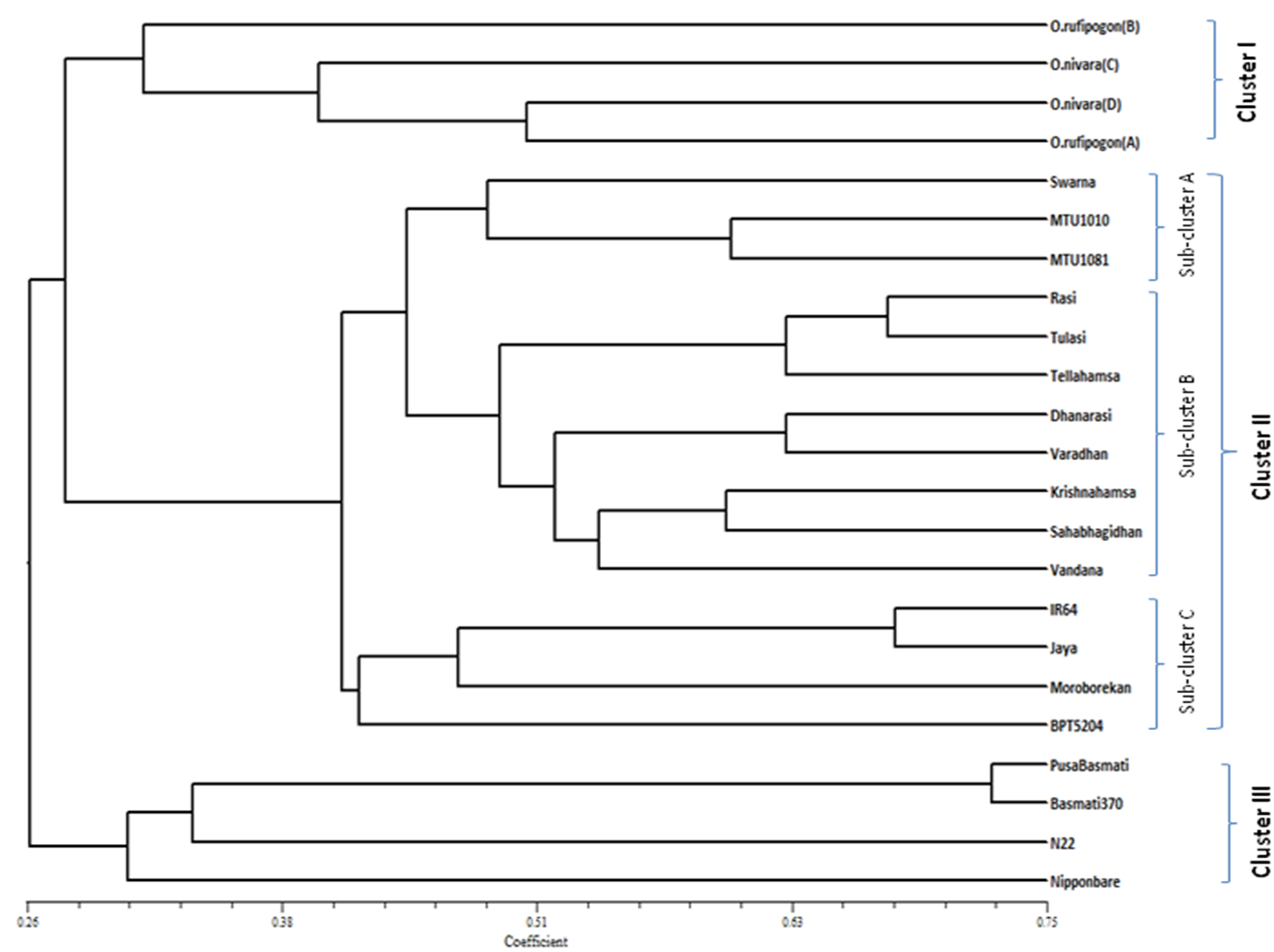** |
| --- |
|  |
